# A comprehensive analysis of metabolomics and transcriptomics to reveal major metabolic pathways and potential biomarkers of human preeclampsia placenta

**DOI:** 10.3389/fgene.2022.1010657

**Published:** 2022-10-03

**Authors:** Yan Feng, Xinlei Lian, Kaimin Guo, Guanglan Zhang, Xuan Huang

**Affiliations:** ^1^ Fetal Care Center, Department of Obstetrics and Gynecology, Guangzhou Women and Children’s Medical Center, Guangzhou Medical University, Guangzhou, China; ^2^ National Risk Assessment Laboratory for Antimicrobial Resistance of Animal Original Bacteria, South China Agricultural University, Guangzhou, China; ^3^ Department of Obstetrics and Gynecology, Guangzhou Women and Children’s Medical Center, Guangzhou Medical University, Guangzhou, China; ^4^ Fetal Medicine Center, Department of Obstetrics and Gynecology, First Affiliated Hospital of Sun Yat-sen University, Guangzhou, China

**Keywords:** preeclampsia, metabolomic, transcriptomic, placenta, metabolic pathways, biomarker

## Abstract

**Background:** The etiology of preeclampsia (PE) remains unclear. With the utilization of metabolomics, dysregulated production of several metabolic components in human plasma, such as lipids, amino acids, androgens and estrogens, was found to be important in the pathogenesis of PE. Transcriptomics adds more in-depth information, and the integration of transcriptomics and metabolomics may yield further insight into PE pathogenesis than either one alone.

**Objectives:** We investigated the placental metabolomics and transcriptomics of PE patients to identify affected metabolic pathways and potential biological targets for exploring the disease pathogenesis.

**Methods:** Integrated transcriptomics and metabolomics were used to analyze five paired human placentas from patients with severe PE and normal pregnancies. This was followed by further validation of our findings in a publicly available dataset of 173 PE vs. 157 control placentas. In addition, weighted gene coexpression network construction was performed to assess the correlation between genetic alterations and diseases.

**Results:** We identified 66 and 41 differentially altered metabolites in negative and positive ion modes, respectively, in the PE group compared to the control group, and found 2,560 differentially expressed genes. Several pathways were aberrantly altered in the PE placenta at both the metabolic and transcriptional levels, including steroid hormone biosynthesis, the cAMP signaling pathway, neuroactive ligand–receptor interactions, taste transduction and prion diseases. Additionally, we found 11 differential metabolites and 11 differentially expressed genes involved in the steroid hormone biosynthesis pathway, indicating impaired metabolism of steroid hormones in the PE placenta. Furthermore, we found that *CYP11A1, HSD3B2*, and *HSD17B6* are highly correlated with diseases.

**Conclusion:** Our findings provide a profile of the dysregulated steroid hormone biosynthesis in PE placenta, we observed a dysregulated cortisol-to-cortisone ratio, testosterone accumulation, decreased testosterone downstream metabolites, impaired production of estrone and estriol, and aberrant hydroxylation and methylation of estradiol. Disorders of placental steroid hormone metabolism might be a consequence or a compensatory change in pathological placentation in PE, which underscores the need to investigate the physiology of steroid hormone metabolites in the etiology of PE.

## 1 Background

Preeclampsia (PE) is a heterogeneous gestational disorder characterized by new-onset maternal hypertension and proteinuria after 20 weeks of gestation, and PE affects approximately 3%–7% of all pregnancies worldwide (Kelly et al., 2017). It can affect virtually every organ system, leading to maternal and fetal morbidity and mortality (Berg et al., 2009; Duley, 2009; Kuklina et al., 2009; Bansil et al., 2010). Abnormal trophoblastic invasion and insufficient remodeling of spiral arteries impair placental perfusion, leading to hypoxia and necrosis, which provoke a cascade of events in the systematic inflammatory response and oxidative stress, eventually resulting in systematic endothelial activation and vasospasm (Rana et al., 2019). Although several theories have been proposed regarding the pathogenesis of preeclampsia, there is no consensus on its etiology or on its early identification and prophylaxis. In recent years, omics approaches have been utilized to understand how it develops and whether we can predict it or treat it, and this has become the focus of numerous studies (Nobakht, 2018; Benny et al., 2020).

Transcriptional profiling or transcriptomics is being increasingly utilized to discover key molecular changes underlying the etiology and early identification of PE (Liu et al., 2018; Tong et al., 2018; Maeda et al., 2019). Metabolic profiling or metabolomics provides data-rich information on metabolic alterations that reflect genetic and epigenetic factors influencing cellular physiology (Nobakht, 2018; Benny et al., 2020). The integration of “-omic” studies may provide a more in-depth understanding of PE pathogenesis (Liu et al., 2013; Leavey et al., 2018; Benny et al., 2020). A metabolomic study showed that several metabolites, such as lipids, amino acids and their derivatives, are promising in the early identification of preeclampsia patients (Nobakht, 2018); moreover, combined approaches of transcriptomics and metabolomics have elucidated altered lipid and amino acid metabolism and dysregulation of the immune response in preeclampsia (Kelly et al., 2017; Shin et al., 2018). In addition, metabolic studies have demonstrated that the mechanism of imbalanced androgen and estrogen production is of great value in the pathogenesis of the disease (Jobe et al., 2013; Shao et al., 2017; Shin et al., 2018; Cantonwine et al., 2019; Shao et al., 2019) and is likely to be associated with alterations in angiogenesis and vascular function (Jobe et al., 2013). Integration of transcriptomics and metabolomics may yield further insight into PE pathogenesis than either approach alone.

**FIGURE 1:**
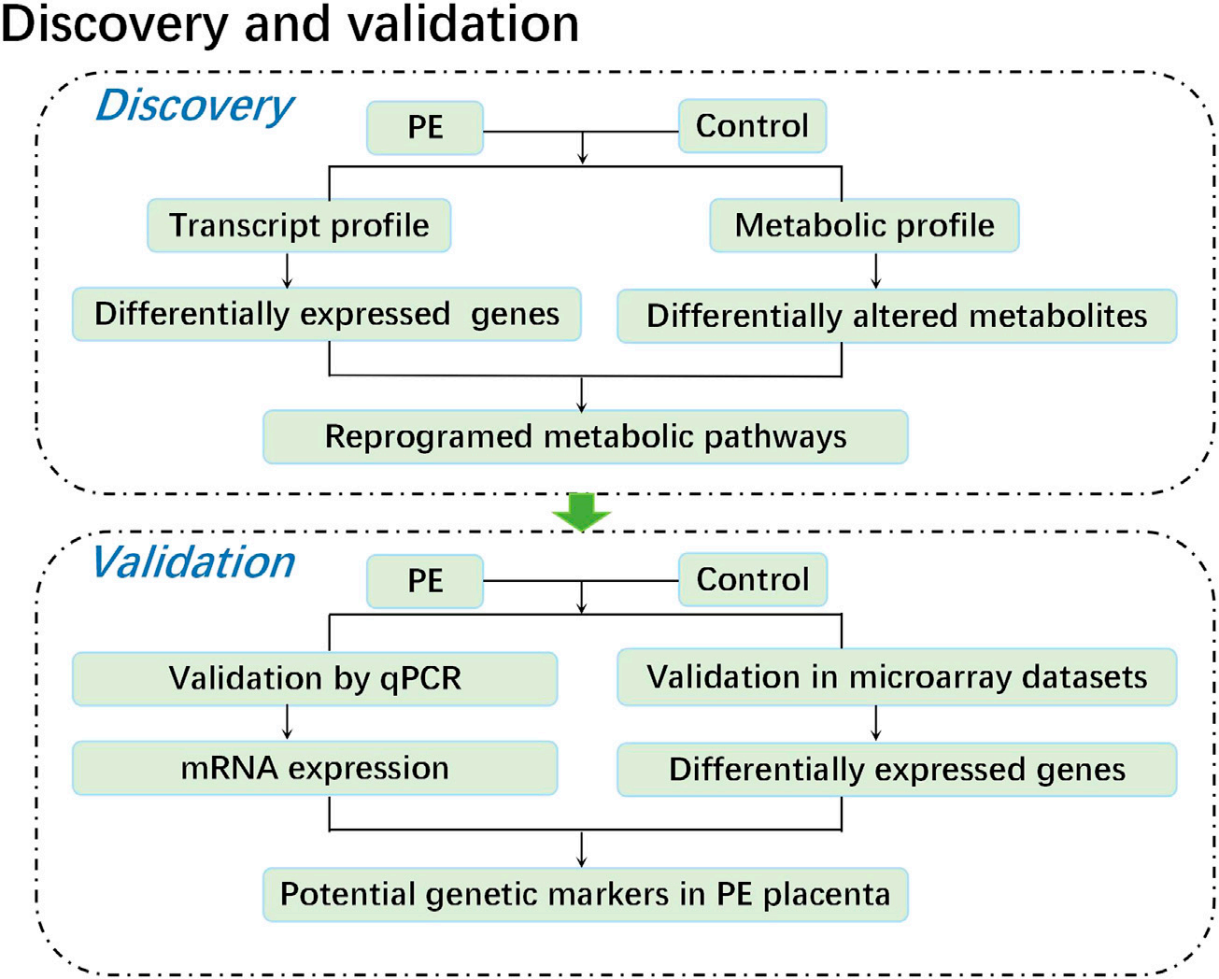
Experimental flow chart.

In this study, we performed a metabolomic study of five paired human placental samples comprising PE placentas and normal pregnancy placentas by liquid chromatography–mass spectrometry (LC–MS), aiming to identify key metabolite alterations unique to human placentas from PE patients. We then performed transcriptome sequencing on these samples to identify genes with significant changes in expression. Through KEGG enrichment analysis, we integrated metabolome and transcriptome data to identify significantly perturbed pathways at both the metabolic and transcriptional levels, revealing potential causes that may aid in understanding the pathogenesis of PE. Finally, these corresponding genetic alterations were further validated by both qPCR data from the same specimens and mRNA expression profile microarray datasets from published studies. Weighted gene coexpression network analysis (WGCNA) was conducted on public mRNA expression profile microarray datasets to assess the correlation between genetic alterations and diseases, providing an effective biological target for exploring the pathogenesis of PE (Figure 1).

## 2 Methods

### 2.1 Study participants

Women aged 20–40 years with singleton pregnancies undergoing selective cesarean section at Guangzhou Women and Children’s Medical Center from January 1 to 31 March 2019, were recruited. The inclusion criteria were as follows: The PE group was included based on the diagnostic criteria described in Gestational Hypertension and Preeclampsia: (ACOG Practice Bulletin 2019). The control group included pregnant women with no preexisting medical diseases or antenatal complications. The exclusion criteria were as follows: pregnancy conceived by assisted reproductive technology; lupus or antiphospholipid antibody syndrome; or placental abruption, chorioamnionitis or meconium-stained fluids. Obtaining samples from qualified controls for early-onset severe PE cases is particularly difficult, since spontaneous late abortion or preterm birth usually result from various pregnancy complications, which are very likely to affect the metabolic state of the placenta. To match the gestational age as closely as possible, we finally included only five severe PE cases (near-term deliveries) and five qualified controls (term deliveries). The included PE cases presented with severe features were given intravenous magnesium sulfate, oral labetalol, and delivered without delay. Preeclampsia was defined as new-onset BP > 140/90 mmHg plus proteinuria (0.3 g/24 h) after 20 weeks in previously normotensive women. Those who met one or more of the following indicators were considered severe cases: 1) systolic blood pressure of 160 mm Hg or more or diastolic blood pressure of 110 mm Hg or more on two occasions at least 4 h apart (unless antihypertensive therapy was initiated before this time), 2) thrombocytopenia (platelet count less than 100 × 10^9^/L), 3) impaired liver function that was not accounted for by alternative diagnoses and as indicated by abnormally elevated blood concentrations of liver enzymes, 4) renal insufficiency (serum creatinine concentration more than 1.1 mg/dl or a doubling of the serum creatinine concentration in the absence of other renal disease), 5) pulmonary edema, 6) new-onset headache unresponsive to medication and not accounted for by alternative diagnoses, and 7) visual disturbances (scotomata, amaurosis fugax, blurred vision, diplopia, homonymous hemianopsia) (Wagner et al., 2011). The institutional review board at the Guangzhou Women and Children’s Medical Center reviewed and approved the study protocol. Written informed consent were obtained from all of the study participants. The clinical and demographic characteristics of the participants included in the study are given in Table 1.

**TABLE 1:** Maternal demographic characteristics and infant outcomes.

	PE	Control	t	*p*
	(n = 5)	(n = 5)		
Maternal age	36.60 ± 6.23	31.60 ± 5.59	1.34	0.219
Gravidity	2.00 (2.00–3.00)	2.00 (1.00–3.00)	-0.67	0.502^a^
Pregestational BMI (kg/m^2^)	22.66 ± 2.41	20.57 ± 2.96	1.22	0.256
Chronic hypertension	0	0	—	—
Gestational diabetes	0	0	—	—
Max systolic pressure (mmHg)	153.40 ± 7.16	114.40 ± 9.42	7.37	<0.001
Max diastolic pressure (mmHg)	97.40 ± 4.72	74.00 ± 8.45	5.40	0.001
Mean gestational age at delivery (weeks)	35.63 ± 1.00	40.05 ± 0.58	-8.56	<0.001
Fetal sex (male)	1 (20.0%)	2 (40.0%)	—	1.000^b^
Birthweight < 10th percentile	3 (60.0%)	0 (0%)	—	0.167^b^

Control, normal pregnancy; PE, preeclamptic pregnancy; NS, not significant. Values are expressed as the means ± SDs. Categorical variables are reported as N (%). Continuous variables were compared between the two groups using a *t* test. Fisher’s exact tests were used to compare categorical variables between the two groups. Note: a indicates that the *p* value was obtained through a nonparametric test; b indicates that the *p* value was obtained through Fisher’s test.

### 2.2 Placental tissue biopsies

After the delivery of the infant, placental tissues were obtained from the maternal central side of the placenta by biopsy forceps at a maximum depth of 0.5 cm. The tissues did not contain the decidual or chorionic plate but had the basal plate and intervillous space and/or placental parenchyma. The specimens were carefully cleaned of visible blood clots before being placed in a cryopreservation tube, quickly frozen in liquid nitrogen, and then transferred to −80°C within 20 min.

### 2.3 Untargeted metabolomic profiling

#### 2.3.1 Metabolite extraction from tissues

One hundred milligrams of tissue was ground with liquid nitrogen, and the homogenate was resuspended in prechilled 80% methanol and 0.1% formic acid by vortexing. The samples were incubated on ice for 5 min and then centrifuged at 15,000 rpm and 4°C for 5 min. A portion of the supernatant was diluted to a final concentration containing 60% methanol by LC–MS grade water. The samples were subsequently transferred to a fresh Eppendorf tube with a 0.22-μm filter and then centrifuged at 15,000 g and 4°C for 10 min. Finally, the filtrate was injected into the LC–MS/MS system for analysis.

#### 2.3.2 UHPLC–MS/MS analysis

LC–MS/MS analyses were performed using a Vanquish UHPLC system (Thermo Fisher) coupled with an Orbitrap Q Exactive HF-X mass spectrometer (Thermo Fisher). Samples were injected onto a Hyperil Gold column (100 × 2.1 mm, 1.9 μm) using a 16-min linear gradient at a flow rate of 0.2 ml/min. The eluents for the positive polarity mode were eluent A (0.1% FA in water) and eluent B (methanol). The eluents for the negative polarity mode were eluent A (5 mM ammonium acetate, pH 9.0) and eluent B (methanol). The solvent gradient was set as follows: 2% B, 1.5 min; 2%–100% B, 12.0 min; 100% B, 14.0 min; 100%–2% B, 14.1 min; and 2% B, 16 min. A Q Exactive HF-X mass spectrometer was operated in positive/negative polarity mode with a spray voltage of 3.2 kV, capillary temperature of 320°C, sheath gas flow rate of 35 arb and aux gas flow rate of 10 arb.

#### 2.3.3 Data analysis

The raw data files generated by UHPLC–MS/MS were processed using Compound Discoverer 3.0 (CD 3.0, Thermo Fisher) to perform peak alignment, peak picking, and quantitation for each metabolite. The main parameters were set as follows: retention time tolerance, 0.1 min; actual mass tolerance, 5 ppm; signal intensity tolerance, 30%; signal/noise ratio, three; and minimum intensity, 100,000. Thereafter, peak intensities were normalized to the total spectral intensity. The normalized data were used to predict the molecular formula based on additive ions, molecular ion peaks and fragment ions. Then, peaks were matched with the mzCloud (https://www.mzcloud.org/) and ChemSpider (http://www.chemspider.com/) databases to obtain accurate qualitative and relative quantitative results. PCA and PLS-DA analysis use R language ropls package and ggbiplot package. The value of Variable Importance in the Projection (VIP) from the first principal component of the PLS-DA model was used, combined with the *p* value of the *t*-test to find differentially expressed metabolites, the threshold was set to VIP > 1.0, and the fold difference FC > 1.5 or FC < 0.667 and *p* value < 0.05. KEGG Pathway was used as the unit and applied the hypergeometric test to find out the Pathways that are significantly enriched in the differential metabolites compared with all the identified metabolite backgrounds.

### 2.4 Transcriptomic profiling

#### 2.4.1 Sample RNA extraction

Five paired placental samples of pregnant women were treated by the following steps. First, total RNA was extracted as previously described (Lian et al., 2016); second, agarose gel electrophoresis was performed to analyze the extent of RNA degradation and contamination; third, Nanodrop was used to assess RNA purity (OD260/280 ratio); fourth, we used Qubit to accurately quantify the RNA concentration; and finally, the Agilent 2,100 Bioanalyzer was used to accurately detect RNA integrity.

#### 2.4.2 Library construction and sequencing

A Ribo-Zero rRNA Removal Kit (Human/Mouse/Rat) (Epicenter, Madison, WI, United States) was used to remove rRNA from total RNA. The library was constructed using an Illumina TruSeq RNA Sample Prep Kit v2 (Illumina Inc., San Diego, CA). Briefly, RNA was sheared into 200–300-bp fragments. In addition, first- and second-strand cDNA was synthesized. Then, short cDNA fragments were purified, end repaired and tailed with a single A (adenine) addition. Adapters were ligated to the A-tailed cDNA fragments. These cDNA fragments were enriched by 12 PCR cycles. Purified libraries were quantified using a Qubit 2.0 Fluorometer (Invitrogen, Carlsbad, CA, United States) and validated using an Agilent 2,100 Bioanalyzer (Agilent, Beijing, China). The reads that passed the Illumina quality filter were kept for sequence analysis.

#### 2.4.3 Data analysis.

High-quality reads were mapped to the human genome by using SOAP aligner/SOAP2 (Li et al., 2009) with five maximum alignment errors. The mRNA abundance was normalized using TPM (Transcripts per million). Differential gene expression analysis was performed by edgeR (edgeR: a Bioconductor package for differential expression analysis of digital gene expression data). Genes with *p* values less than 0.05 and fold changes > 1.5 or < 0.667 were considered differentially expressed genes (DEGs). Gene list enrichment analysis used Enrichr online tools (https://maayanlab.cloud/Enrichr/).

#### 2.4.4 Public dataset acquisition

The mRNA expression profile microarray dataset was from Gene Expression Omnibus (GEO), NCBI’s publicly available genomics database, and the data number is GSE75010. Next, we performed differential analysis (adjusted *p* value < 0.05, and fold changes > 1.5 or < 0.667) by comparing PE placentas to normal pregnancy placentas in the R computing environment using the limma package. Weighted gene coexpression network construction was performed by the WGCNApackage in R as previously described (Zhang and Horvath, 2005; Langfelder and Horvath, 2008). The expression of the genes for all samples were inputted to construct the weighted gene coexpression network.

### 2.5 RT-qPCR

The qRT-PCR was performed with SYBR Premix Ex Taq (Takara, Dalian, China) in an iQ5 thermal cycler (Biorad, Hercules, CA) according to the manufacturer’s instructions. The cycling conditions were as follows: 94°C for 5 min, followed by 35 cycles at 94°C for 1 min, at 55°C for 1 min and 72°C for 1 min with a final step of 72°C for 5 min. Melting curves were read from 60 to 95°C in steps of 1°C. Normalized expression levels of the target gene transcripts were calculated relative to GAPDH. Then *t*-test was used to RT-qPCR statistical analysis. The primer information is listed in Supplementary Table S9.

## 3 Results

### 3.1 Demographic characteristics and infant outcomes

Five PE cases and five maternal age-matched controls were included in the study. The maternal demographic characteristics and infant outcomes of the PE cases and controls are presented in Table 1. Fetal birth weight and gestational age at delivery were lower in preeclamptic pregnancies than in normotensive pregnancies.

### 3.2 Metabolomic and transcriptional analysis revealed the major pathways that are altered in PE

Ten placental specimens from PE and normotensive controls were analyzed by liquid chromatography–mass spectrometry (LC–MS/MS) using a Vanquish UHPLC system coupled with an Orbitrap Q Exactive HF-X mass spectrometer. Principal component analysis (PCA) and partial least squares discrimination analysis (PLS-DA) were carried out, as shown in Supplementary Figure S2. A total of 980 metabolites were identified, and the relative changes in metabolites were compared between the two groups. Fold changes and *p* values are presented. This approach yielded 66 differentially altered metabolites in negative ion mode (variable importance in projection, VIP > 1, *p* < 0.05, fold change, FC > 1.5 or FC < 0.667), among which 49 were downregulated and the remaining metabolites were upregulated. In positive ion mode, there were 41 differentially altered metabolites, of which 15 were downregulated and the rest were upregulated (Supplementary Table S1).

**FIGURE 2:**
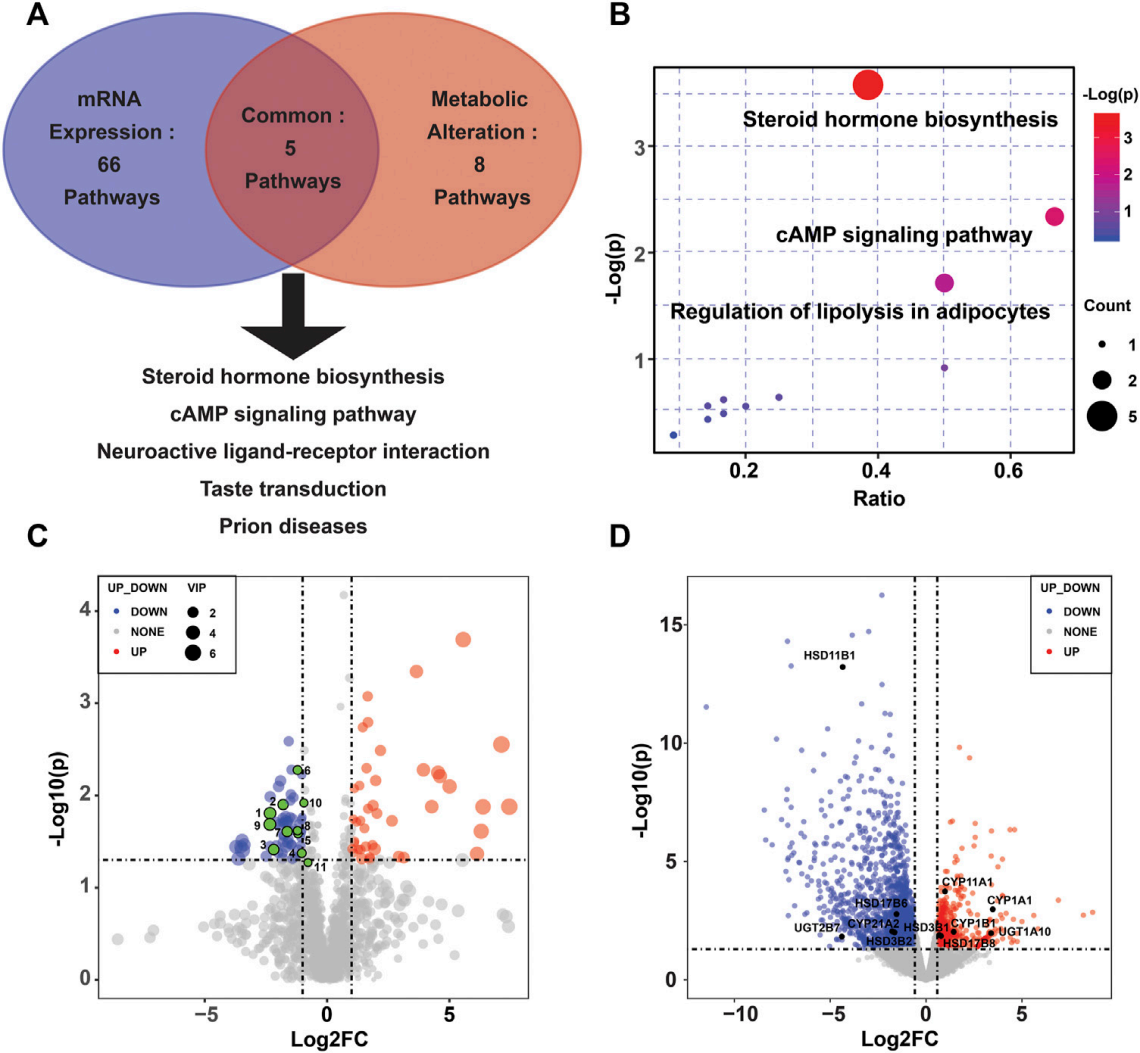
Altered pathways in the PE placenta. **(A)** Metabolic and transcriptional analysis identification of five pathways that were significantly altered in PE placentas. **(B)** Overview of the pathway analysis based on metabolite alterations. **(C)** Volcano plots of the differential metabolites in the steroid hormone biosynthesis pathway. **(D)** Volcano plots of the differentially expressed genes in the steroid hormone biosynthesis pathway.

To reveal the enzymes involved, transcriptional profiling of the same specimens was conducted to discover DEGs. Of the 2,560 significantly DEGs (*p <* 0.05, FC ≥ 1.5) (Supplementary Table S2), 627 were upregulated, and the remaining genes were downregulated. Using KEGG pathway significant enrichment analysis, five pathways were identified to be significantly altered at both the metabolomic and mRNA expression levels in the PE group (Figure 2A, Supplementary Tables S3,S4), including the metabolism of steroid hormone biosynthesis, cAMP signaling pathway, neuroactive ligand–receptor interaction, taste transduction and prion diseases. Figure 2B demonstrates the altered pathways based on metabolic profiling, among which we identified steroid hormone biosynthesis as the metabolic pathway of greatest interest, as it exhibited lower *p* values and the most enriched differential metabolites. There were 11 differential metabolites and 11 DEGs involved in the steroid hormone biosynthesis pathway (Figures 2C,D).

### 3.3 Alterations in metabolite and mRNA expression in the steroid hormone biosynthesis pathway in the PE placenta

Steroid hormones are all derived from cholesterol and are metabolized into glucocorticoids, mineralocorticoids, and sex hormones mainly through the cytochrome P450 (*CYP*) family and the hydroxysteroid dehydrogenase (*HSD*) family (Beaudoin et al., 1997). We found alterations in glucocorticoids and sex hormones at the metabolic and transcriptomic levels in the present study, and the relative levels of metabolites of interest and gene mRNAs are highlighted in Supplementary Tables S2,S3.

**FIGURE 3:**
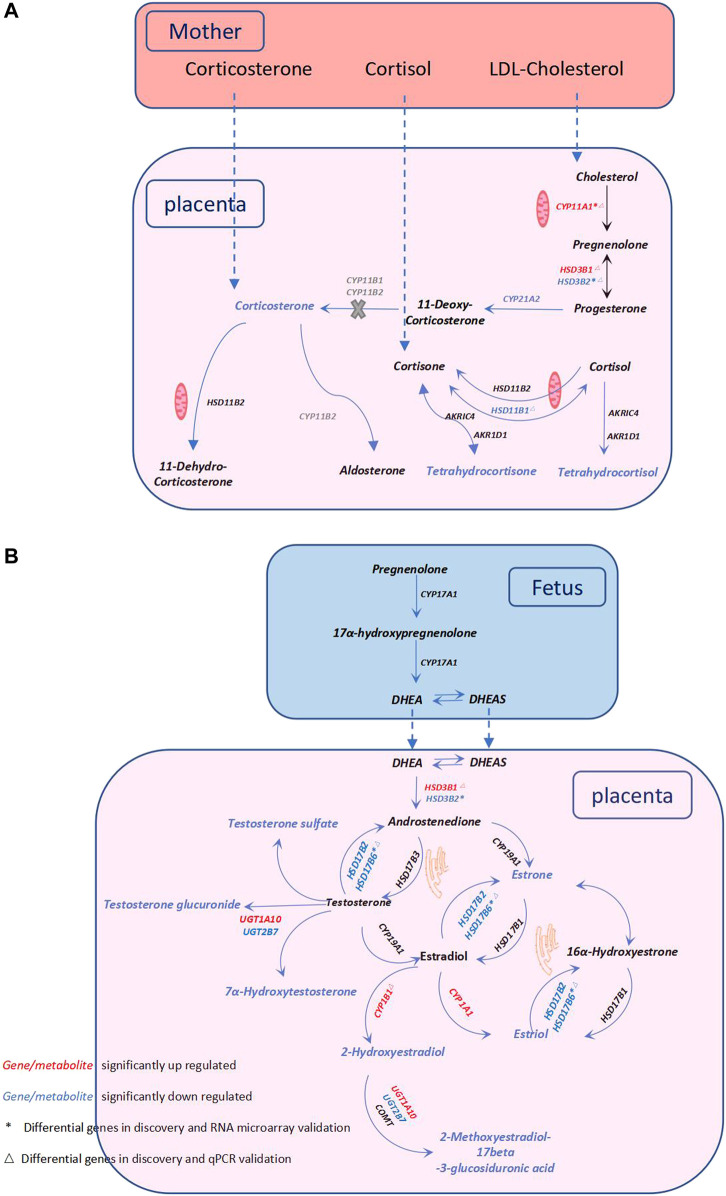
Metabolic pathway alterations in PE—Steroid hormone biosynthesis. **(A)** Description of glucocorticoid metabolism. **(B)** Description of sex hormone metabolism. Steroid hormones are synthesized from cholesterol mainly through the catalysis of the cytochrome P450 (*CYP*) family and the hydroxysteroid dehydrogenase (*HSD*) family. The catalysis of cholesterol is initiated by *CYP11A1*, and cholesterol is subsequently metabolized into glucocorticoids, mineralocorticoids, and sex hormones. The dysregulation of glucocorticoid metabolism and sex hormones in the PE placenta is illustrated in **(A)** and **(B)**, respectively. Red and blue fonts indicate significant increases and decreases, respectively. * Differential genes in the discovery and RNA microarray validation. △ Differential genes in the discovery and qPCR validation. The genes in grey represent they are lack in the placenta, and the X in grey indicates that this reaction is blocked in the placenta.

Regarding glucocorticoid metabolism, PE placentas exhibited a significant reduction in 17α-hydroxypregnenolone, corticosterone, tetrahydrocortisol, and tetrahydrocortisone compared to control placentas. A lower placental cortisol/cortisone ratio was observed in the PE group, but the difference was not significant (Table 2). Furthermore, we examined the corresponding genes involved in glucocorticoid biosynthesis. Compared with the controls, upregulated *CYP11A1* and *HSD3B1* and decreased *HSD3B2, HSD11B1*, and *CYP21A2* were observed in the PE group. *CYP11A1*, a key enzyme initiating the catalysis of cholesterol, was significantly upregulated in PE. *HSD3B*, which has two isoforms (*HSD3B1* and *HSD3B2*), is a rate-limiting enzyme involved in the conversion of 17α-hydroxypregnenolone to 17α-hydroxyprogesterone. *HSD3B1* was significantly upregulated, while *HSD3B2* was downregulated. *CYP21A2* plays a key role in the transformation of progesterone to corticosterone and exhibited a significant reduction. *HSD11B1* and *HSD11B2* are vital to proper cortisol metabolism. *HSD11B2* catalyzes the metabolization of cortisol to cortisone and protects the fetus from exposure to excessive maternal cortisol during pregnancy, whereas *HSD11B1* performs the reverse function of *HSD11B2* and reactivates cortisol by converting cortisone to cortisol (Berkane et al., 2018). We observed significantly decreased *HSD11B1*, nonsignificantly elevated *HSD11B2*, and a lower cortisol/cortisone ratio in PE cases. These findings are summarized in Figure 3A.

**TABLE 2:** Activity of the main placental enzymes involved in steroidogenesis in the PE and control groups.

*Enzyme involved*	PE	Control	*p* value
*Metabolite’s ratio*	(n = 5)	(n = 5)	
*HSD11B1/HSD11B2*			
*Cortisol/Cortisone*	2.64e-2	5.05e-2	0.193
*HSD17B8/HSD17B6*			
*T/A4*	4.39	1.22	0.154
*CYP19A1*			
*E1/A4*	48.77	54.39	0.421

Control, normal pregnancy; PE, preeclamptic pregnancy; NS, not significant. Values are expressed as the means ± SDs. E1, estrone; A4, androstenedione.

With regard to sex hormone biosynthesis, PE exhibited no significant increase in testosterone in the PE placenta and a significant decrease in testosterone metabolites such as testosterone sulfate, testosterone glucuronide, and 7α-hydroxytestosterone. In addition, we found significantly decreased estradiol metabolites, including estrone (E1) (VIP = 0.919), estriol (E3) (VIP = 0.986), 2-hydroxyestradiol and 2-methoxyestradiol-17beta-3-glucosiduronic acid. Genes of this pathway were enriched in the PE placenta. *HSD17B6* (an isoform of *HSD17B*) was significantly downregulated, and the corresponding testosterone (T)/androstenedione (A4) ratio was found to be increased (Table 2). *CYP1A1* and *CYP1B1* were significantly increased in PE; however, the corresponding downstream metabolites E3 and 2-hydroxyestradiol were significantly decreased. *CYP19A1* in the PE group was not significantly altered, nor was the corresponding E1/A4 ratio (Table 2). *UGT1A10* was found to be increased; conversely, *UGT2B7* was found to be decreased, while the glucuronide forms of testosterone and 2-hydroxyestradiol were significantly decreased in PE. All alterations at both the metabolic and transcriptional levels in this pathway are illustrated in Figure 3B.

### 3.4 Validation of the metabolomic and transcriptomic alterations in PE

**FIGURE 4:**
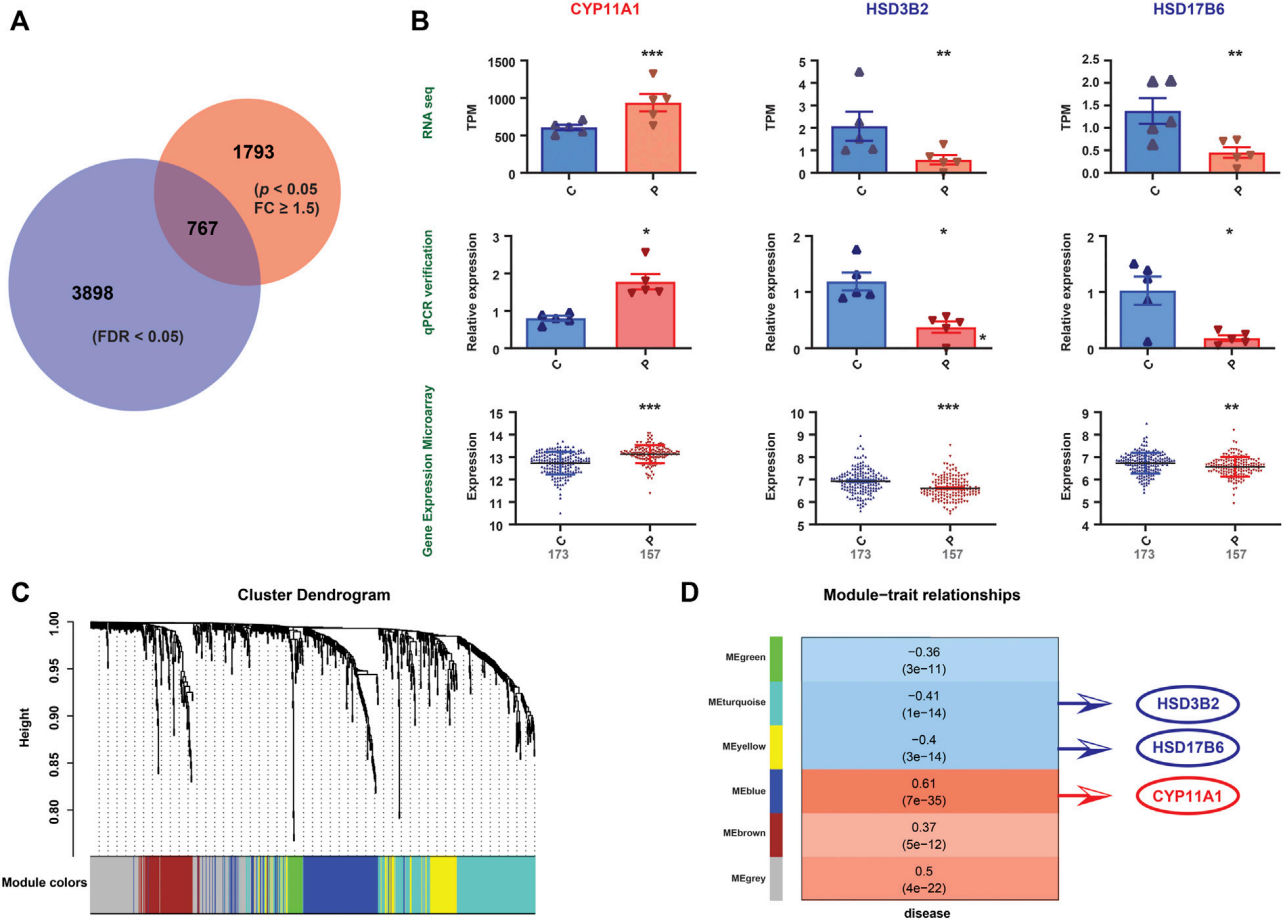
Validation of the gene expression patterns. **(A)** Differential gene expression between the PE and control groups from RNA-seq data (indicated by the red circle). Differential gene expression between the PE and control groups from mRNA expression profile microarray datasets (indicated by the blue circle). The Venn diagram represents the intersection between the two sets (767 genes). **(B)** Among the 767 genes, three differentially expressed genes (*CYP11A1, HSD3B2*, and *HSD17B6*), which correlate with steroid hormone biosynthesis, were altered uniformly in the RNA-seq data, qPCR validation and mRNA expression profile microarray datasets. Significant differences, *: *p <*0.05, **: *p <*0.01, ***: *p <*0.001. **(C)** The figure demonstrates the differentially expressed gene cluster dendrogram analysis in the preeclampsia vs. control groups. The five colors, namely, green, turquoise, yellow, blue, and brown, represent the modules. The dynamic tree cut method was implemented for analysis. The degree of coexpression between the genes assigned by the same module was relatively high. **(D)** The figure represents the correlation between mRNA module eigengenes and phenotypic traits. Each row represents the module eigengene or ME (ME is the correlation matrix of the module and sample, labeled by color), and each column represents a trait. Each square contains Pearson’s correlation coefficient between the MEs and traits and their associated *p* values. The red and blue colors show a strong positive and negative correlation, respectively. The *HSD17B6*, *HSD3B2* and *CYP11A1* genes are located in three modules, that is, turquoise, yellow, and blue, respectively. These three modules are highly correlated with diseases.

To validate our findings, qPCR was conducted on the same specimens, and we revealed consistent alterations between the transcriptional analysis and qPCR-quantified mRNA expression levels (Supplementary Figure S1). Furthermore, we validated the transcriptional findings based on the mRNA expression profile microarray datasets of 330 human placenta samples from eight previously published studies (Leavey et al., 2016), including 173 preeclamptic and 157 control placentas. A total of 2,650 DEGs were found in our RNA-seq dataset (*p* < 0.05, FC ≥ 1.5) (Supplementary Table S2), while 4,665 were found in the mRNA expression profile microarray dataset (FDR<0.05) (Supplementary Table S5), among which 767 were identified to be altered in both datasets (Figure 4A). The comparisons of the enriched genes in the steroid hormone biosynthesis pathway between these two datasets are shown in Supplementary Table S8. Furthermore, three genes (*CYP11A1, HSD3B2*, and *HSD17B6*) involved in the steroid hormone biosynthesis pathway were consistently altered in the RNA-seq data, publicly available mRNA expression profile microarray datasets, and qPCR validation (Figure 4B). In addition, we conducted WGCNA. Five modules were constructed. The red and blue colors showed strong positive and negative correlations, respectively. *HSD17B6* and *HSD3B2* were strongly negatively correlated with diseases, and *CYP11A1* presented a strong positive correlation. These genes were located in three modules, namely, turquoise, yellow, and blue. These three modules were highly correlated with diseases (Figures 4C,D, Supplementary Table S6).

## 4 Discussion

In the present study, we performed a comprehensive analysis based on metabolic and transcriptomic data and found a metabolic reprogramming in the PE placenta. The multi-omics analysis in this study found five pathways that were significantly altered in the PE placenta compared to the control, and steroid hormone biosynthesis was identified as the most significantly enriched metabolic pathway and was further investigated. *CYP11A1* encodes the P450scc enzyme that catalyzes the first and rate-limiting step of steroid biosynthesis (Hu et al., 2004), converting cholesterol to pregnenolone, which is further metabolized into three major steroid hormone classes: mineralocorticoids, glucocorticoids, and sex steroids. The main corresponding enzymes involved in this metabolic process include *HSD3B, CYP17, CYP21, CYP11B, HSD11B, HSD17B*, and *CYP19* (Shih et al., 2011). To date, the pathogenesis of PE is poorly understood, and defective placentation is thought to be causal. Dysregulation of sex hormone metabolism plays a critical role in PE placental dysplasia by affecting trophoblast differentiation and uterine-placenta angiogenesis (Morales et al., 1995; Maliqueo et al., 2016; Berkane et al., 2017; Berkane et al., 2018). Our findings provide a profile of the metabolic and genetic changes in the PE placenta, as well as the alterations in steroid hormone biosynthesis at both the metabolic and transcriptional levels in the PE placenta.

### 4.1 Initiation of steroid hormone biosynthesis—conversion from cholesterol to progesterone

Steroid hormones are initially synthesized from cholesterol, which is essentially taken up from the maternal circulation. In the first step, the conversion of cholesterol to pregnenolone was catalyzed by cytochrome P450scc (*CYP11A1*) in mitochondria. Subsequently, pregnenolone is converted to progesterone by *HSD3B* (*HSD3B1* and *HSD3B2*) (Hu et al., 2004; Shih et al., 2011).

In the present study, we found that *CYP11A1* expression is upregulated in the PE placenta compared to that of the control group. We validated this finding and demonstrated that *CYP11A1* presents a strong positive correlation with PE (Figure 4B). One study reported overexpression of *CYP11A1* in preeclamptic maternal serum with cotemporally increased serum pregnenolone and progesterone concentrations (Moon et al., 2014). In the placenta, *CYP11A1* is located in syncytial trophoblasts (Hill et al., 2014). Upregulated *CYP11A1* enhances trophoblast autophagy and inhibits trophoblastic invasion, which is associated with the etiology of PE (Pan et al., 2017). Yu Chien et al. established a *CYP11A1* transgenic mouse model and found that overexpression of *CYP11A1* induces the loss of decidual cell polarity and results in anomalous placentation (Chien et al., 2013). Placentation defects are leading causes of PE, and *CYP11A1* might play a critical role in placental dysplasia *via* oxidative stress and hypoxia.

*HSD3B* is a key rate-limiting enzyme in steroid biosynthesis pathways. It is expressed as two tissue-specific isoforms (*HSD3B1* and *HSD3B2*) (Thomas et al., 2005). *HSD3B1* is almost exclusively expressed in the placenta and peripheral tissues, while *HSD3B2* is predominantly expressed in the adrenal gland, ovary, and testis (Simard et al., 1996). *HSD3B2* is a bifunctional enzyme that plays a key role in the conversion of pregnenolone to progesterone and DHEA to A4. In our study, we found significantly upregulated *HSD3B1* and downregulated *HSD3B2* in PE but confirmed only the alteration in *HSD3B2* in the validation course, no difference in *HSD3B1* expression between PE and the controls was found in the validation sets (Leavey et al., 2016), finding that *HSD3B2* was highly negatively correlated with disease in WGCNA (Figure 4D). The reduction in *HSD3B2* might play a role in the dysregulated synthesis of progesterone in the PE placenta. However, the low placental expression of *HSD3B2* and the expression difference of *HSD3B1* between RNA-seq and validation microarray data sets bring concerns, thus confirming *HSD3B1, HSD3B2* and their downstream proteins at protein level may provide more valuable information.

### 4.2 Metabolic profile of glucocorticoid biosynthesis in the preeclamptic placenta

Transplacental transfer of biologically active glucocorticoids is unidirectionally catalyzed by *HSD11B2*, which converts corticosterone to 11-dehydrocorticosterone and cortisol to cortisone. *HSD11B1* catalyzes the reverse reaction to *HSD11B2*, and it reactivates cortisol by converting cortisone to cortisol (Causevic and Mohaupt, 2007; Jayasuriya et al., 2019). These conversions inactivate cortisol and corticosterone, thereby protecting the fetus from exposure to high levels of active glucocorticoids. This exposure may lead to gestational hypertension (Siiteri and MacDonald, 1966; Staud et al., 2006; Abbassi-Ghanavati et al., 2009; Aufdenblatten et al., 2009; Jayasuriya et al., 2019). In PE, enhanced systematic activity of maternal *HSD11B2* or reduced *HSD11B1* activity were demonstrated, which is expected to result in a decreased maternal serum cortisol-to-cortisone ratio (Jayasuriya et al., 2019). However, other studies came to the opposite conclusion, showing reduced placental *HSD11B2* expression in PE and paradoxically indicated that reduced *HSD11B2* activity would be expected to result in increased maternal cortisol-to-cortisone ratios (McCalla et al., 1998; Schoof et al., 2001; Hu et al., 2015). In the present study, we demonstrated significantly reduced *HSD11B1* and nonsignificantly increased *HSD11B2* at the transcriptional level and a decreased cortisol-to-cortisone ratio in the PE placenta. This might be a placenta-specific compensatory response to increased fetal exposure to systematic cortisol, resulting from the increased transplacental transfer of cortisol in preeclampsia patients, but this hypothesis requires further confirming.

### 4.3 Metabolic profile of androgen and estrogen biosynthesis in the preeclamptic placenta

The conversion from pregnenolone to dehydroepiandrosterone (DHEA), dehydroepiandrosterone sulfate (DHEAS), and 16OH-DHEAS was accomplished in the fetal or maternal adrenal gland and liver. The human placenta lacks *CYP17A1* (Christensen, 1974), but it does express *HSD17B, CYP19A1*, and *HSD3B* (Mason et al., 1993; Takeyama et al., 1998). Therefore, the placenta can synthesize A4 from fetal DHEAS and 16OH-DHEAS and undertake the subsequent synthesis of both testosterone and estradiol (Morel et al., 2016).

High circulating testosterone and aberrant estrogen synthesis in PE were observed in previous studies (Zhorzholadze et al., 2006; Shao et al., 2017; Kumar et al., 2018; Keya et al., 2019; Shao et al., 2019; Lan et al., 2020). Augmented placental androgen synthesis and decreased placental aromatase expression in PE were observed in recent studies (Hertig et al., 2010); however, the role of placenta-derived androgen and estrogen in preeclampsia remains elusive. We observed the T to A4 ratios in the PE and control groups were 4.39 and 1.22, respectively (*p* = 0.154), whereas the production of testosterone derivatives, such as testosterone sulfate, testosterone glucuronide, and 7α-hydroxytestosterone, were significantly decreased. Moreover, the production of metabolites derived from estradiol (E2), including E1, E3, 16α-hydroxyestrone, 2-hydroxyestradiol and 2-methoxyestradiol-17beta-3-glucosiduronic acid were likely to be impaired. These findings may partially explain the accumulation of testosterone and the impaired production of estrogens in PE placenta.

*HSD3B* were involved in the conversion of DHEA to A4 (Berkane et al., 2017). In the present study, increased *HSD3B1* and decreased *HSD3B2* in PE were demonstrated. The effect of *HSD3B* on androgen metabolism in the PE placenta is controversial. Hogg, K demonstrated significant hypomethylation of *HSD3B1* in the placenta of early-onset preeclampsia (Hogg et al., 2013). Shimodaira, M showed that polymorphisms of maternal *HSD3B1* and *HSD3B2* were not associated with preeclampsia (Shimodaira et al., 2012). Therefore, the protein expression of *HSD3B1, HSD3B2* in PE placenta, and the role of *HSD3B* in abnormal androgen synthesis in the placenta of PE remains intriguing unsolved mysteries.

*HSD17B* plays a role in the conversion of A4 to T and E1 to E2, it has multiple isoforms (*HSD17B1* to *HSD17B8*). Shao, X found that *HSD17B3* is markedly increased in PE placenta and that it is the key enzyme that converts A4 to T (Shao et al., 2017). However, other researchers claimed that the activity of *HSD17B3* in the preeclamptic placenta was compromised (Shin et al., 2018; Shao et al., 2019). In our study, a 0.43-fold elevation in *HSD17B3* expression was found in PE placentas (*p* = 0.64). In the validation datasets of 330 human placental samples, placental *HSD17B3* increased 0.03-fold in the PE group (*p* = 0.63). These findings indicated that *HSD17B3* did not change significantly at the transcriptional level in the PE placenta; however, the status of its posttranscriptional modification, protein expression, and enzyme activity in PE remains to be elucidated. Conversion of E1 to E2 in the placenta depends on the presence of *HSD17B1* (Husen et al., 2003), and it has been reported to be an independent risk factor for predicting PE (Ishibashi et al., 2012; Ohkuchi et al., 2012). The transcriptional level of *HSD17B1* was not altered significantly in our study; however, it demonstrated a 0.51-fold reduction in the PE group in the validation dataset analysis (*p* = 2.52E-21). Decreased placental *HSD17B1* expression might partly explain the reduction in E2 in PE patients. In addition, *HSD17B2* and *HSD17B6* were noticeably decreased in PE placentas in the present study (*p* = 0.179 and *p* = 0.002, respectively). These two genes were observed to decrease markedly in the PE group in the validation datasets (*p* = 4.41E-10 and *p* = 0.007, respectively). *HSD17B2* and *HSD17B6* perform the reverse reactions of isoforms 1 and 3, converting E2 into E1 and A4 into T, respectively (Saloniemi et al., 2012). Together with other studies, we highlight a possible correlation between the reduction in *HSD17B2* and *HSD17B6* and the elevated T level in PE. Further studies are warranted to validate these findings and to reveal the underlying mechanism.

*CYP19A1* converts A4 into E1 and T into E2. In several studies, the protein and mRNA levels of placental aromatase were significantly decreased in PE placentas compared to normotensive pregnancies. Accordingly, the ratios of the corresponding metabolites, that is, the circulating concentrations of 17-β- E2/T and E1/A4, were significantly decreased in PE patients (Hertig et al., 2010; Perez-Sepulveda et al., 2015; Zhang et al., 2016; Berkane et al., 2018). However, our observations demonstrated that placental *CYP19A1* was not altered significantly at the transcriptional level (*p* = 0.42), which was confirmed in the analysis of validation datasets of 330 human placental samples (*p* = 0.60). E1/A4 was reduced in the PE group (48.77 vs. 54.93, *p* = 0.42) in our research, and similarly, it was found to decrease significantly in another study (Berkane et al., 2018), suggesting a deficiency of placental aromatase in preeclampsia, which might not be at the transcriptional level but rather at the level of posttranscriptional modification and regulation.

### 4.4 Possible connections between the pathogenesis of PE and the aberrant placental synthesis and metabolism of steroid hormones

**FIGURE 5:**
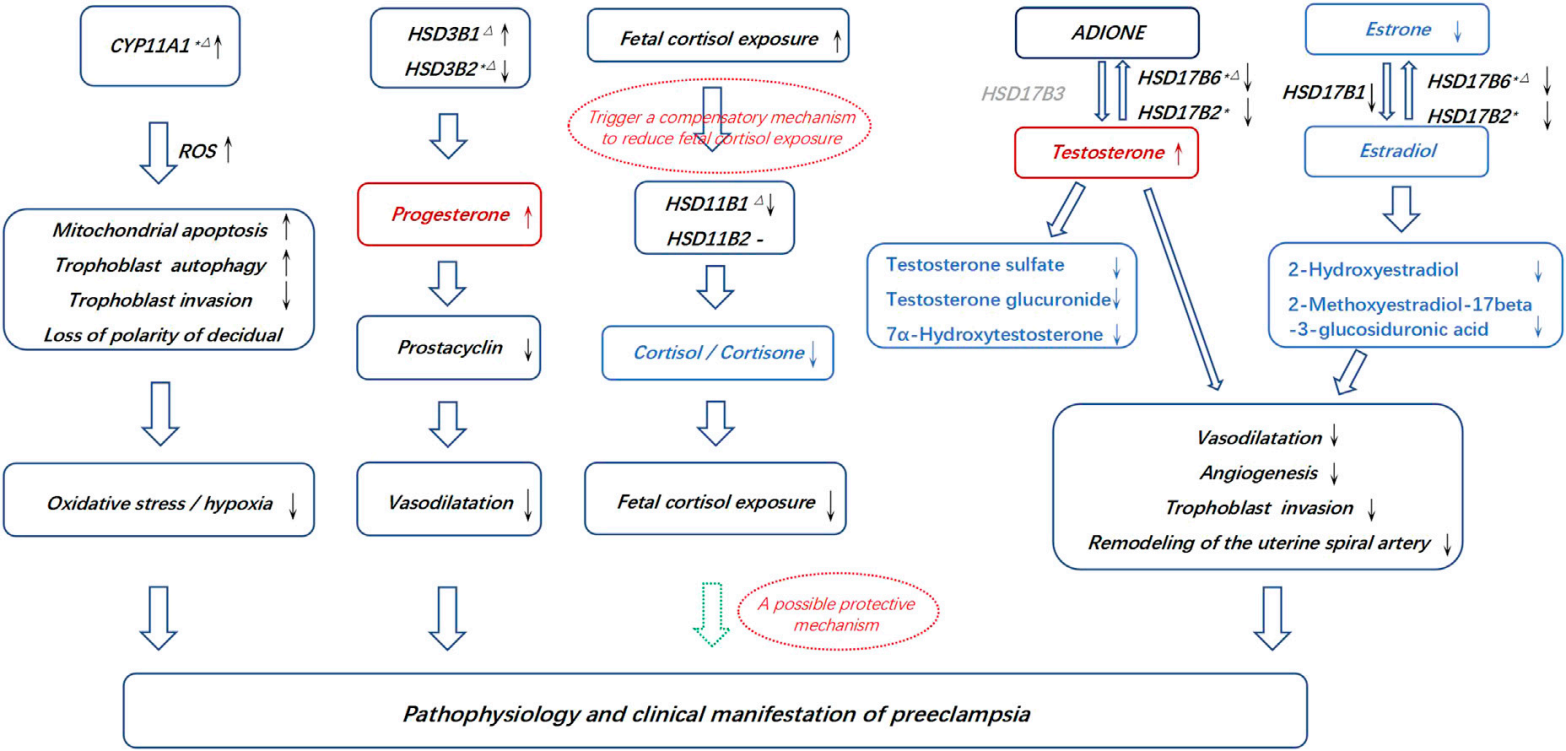
Possible connections between the pathogenesis of PE and the aberrant placental synthesis and metabolism of steroid hormones. * Differential genes in the discovery and RNA microarray validation. △Differential genes in the discovery and qPCR validation. The dotted hollow arrow in green represents a potential protective effect. The metabolites in red represents an accumulation, and the metabolites in blue represents aberrantly affected biosynthesis. The gene in grey represents that it is lack in placenta.

Increased trophoblast progesterone in PE may act by a paracrine mechanism to inhibit prostacyclin synthesis in the placental vasculature, leading to compromised vasodilatation (Walsh and Coulter, 1989). Testosterone has been shown to be associated with the inhibition of angiogenesis at the fetal-maternal interface and increases uterine vascular constriction (Baker et al., 1978; Blesson et al., 2015; Maliqueo et al., 2016). However, estrogens and their metabolites act as vasodilators, leading to an increase in uterine blood flow, promoting early placental angiogenesis and regulating the remodeling of the uterine spiral artery by the invasion of placental trophoblast cells (Jobe et al., 2013; Lechuga et al., 2015; Shao et al., 2017). Inactivation of cortisol and corticosterone may protect the fetus from exposure to high levels of active glucocorticoids in gestational hypertension (Siiteri and MacDonald, 1966; Staud et al., 2006; Abbassi-Ghanavati et al., 2009; Aufdenblatten et al., 2009; Jayasuriya et al., 2019). Thus, we propose a working hypothesis that disrupted steroid hormone production in preeclampsia may lead to insufficient vascular dilation, enhanced oxidative stress or hypoxia, compromised angiogenesis, insufficient trophoblast invasion and uterine spiral artery remodeling, thereby triggering some of the pathophysiology of preeclampsia (Figure 5). These metabolic disorders might be consequent or compensatory changes in pathological placentation in PE. The above alterations in the PE placenta may lead to corresponding changes in maternal plasma and underscore the need for prospective longitudinal studies covering the first, second, and third trimesters to further assess and validate whether these metabolites and genes involved in steroid hormone biosynthesis can be useful biomarkers in the early detection of PE and to understand the pathophysiology of the disease.

### 4.5 Limitations

The sample size of this research was limited. The validation microarray datasets showed mild difference in the expressions of *CYP11A1*, *HSD3B2*, and *HSD17B6* between PE and control groups, compared to mRNA seq. The possible reasons might be that, these data were generated from seven independent researches, participants were from different races, the gestational age at delivery varied widely, but the three genes showed the same trend of change in different researches. Furthermore, we failed to match each case with controls of the same gestational age. The reason for not including gestational age-matched preterm delivery patients into the control group is that they are often complicated by chorioamnionitis, placental abruption, fetal growth restriction with fetal distress, maternal-fetal hemorrhage, *etc.*, which were likely to affect the metabolic state of the placenta. Another concern is that the observed transcriptional differences might not lead to corresponding protein or functional difference. Further investigations should aim to identify these genes and their downstream proteins at the protein level and explore their functions.

## 5 Conclusion

In summary, the perturbed metabolism in PE placenta affected a diverse array of intracellular pathways, including steroid hormone biosynthesis, the cAMP signaling pathway, neuroactive ligand–receptor interaction, taste transduction and prion diseases. Steroid hormone biosynthesis was the most significantly enriched metabolic pathway. A dysregulated cortisol-to-cortisone ratio, accumulation of testosterone, impaired estrogen synthesis, and aberrant hydroxylation and methylation of estradiol were observed in PE placentas. DEGs identified in this metabolic process could be the cornerstone of the dysregulated steroid hormone biosynthesis in severe PE placentas. However, the disorders of placental steroid hormone metabolism found in the present study may also be a consequence or a compensatory change in the pathological placentation of PE. Prospective longitudinal studies covering the first, second, and third trimesters are needed to further assess and validate these conclusions.

## Data Availability

The datasets presented in this study can be found in online repositories. The names of the repository/repositories and accession number(s) can be found below: https://ngdc.cncb.ac.cn/gsa-human/s/0m207aH0, PRJCA0098 67.

## Abbreviations

A4, Androstenedione; BMI, Body Mass Index; CYP, Cytochrome P450; DHEAS, Dehydroepiandrosterone Sulfate; DEGs, Differentially Expressed Genes; DHEA, Dehydroepiandrosterone; E1, Estrone; E2, Estradiol; E3, Estriol; FC, Fold Change; GEO, Gene Expression Omnibus; HSD, Hydroxysteroid Dehydrogenase; LC–MS, Liquid chromatography–mass spectrometry; NS, Not Significant; PCA, Principal Component Analysis; PLS-DA, Partial Least Squares Discrimination Analysis; PE, Preeclampsia; SGA, Small for Gestational Age; T, Testosterone; VIP, Variable Importance in the Projection; WGCNA, Weighted Gene Coexpression Network Analysis.
