# Supplementary material for: A comprehensive analysis of metabolomics and transcriptomics to reveal major metabolic pathways and potential biomarkers of human preeclampsia placenta
Source: Front Genet. 2022 Oct 3;13:1010657. doi: 10.3389/fgene.2022.1010657 (PMC9574103; doi:10.3389/fgene.2022.1010657)
Supplement: Supplementary file 1 [file DataSheet4.docx]

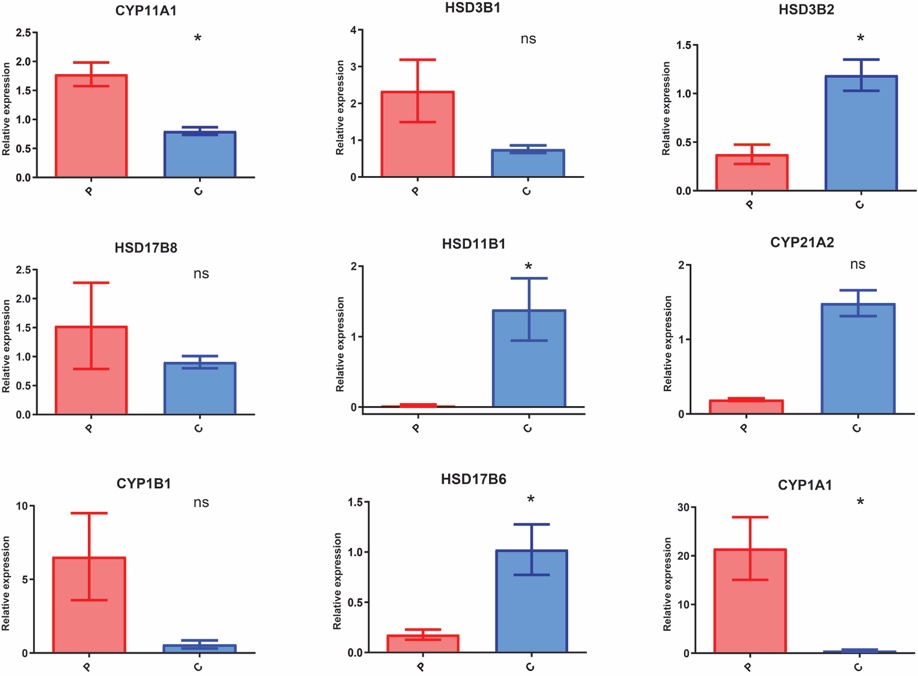


Supplemental Figure 1**. Validation of gene expression by qPCR.** Histogram of nine important genes in the placenta by qPCR validation. The red and blue [histogram](http://dict.youdao.com/w/histogram/#keyfrom=E2Ctranslation)s represent the PE and control groups, respectively. * *p*＜0.05.


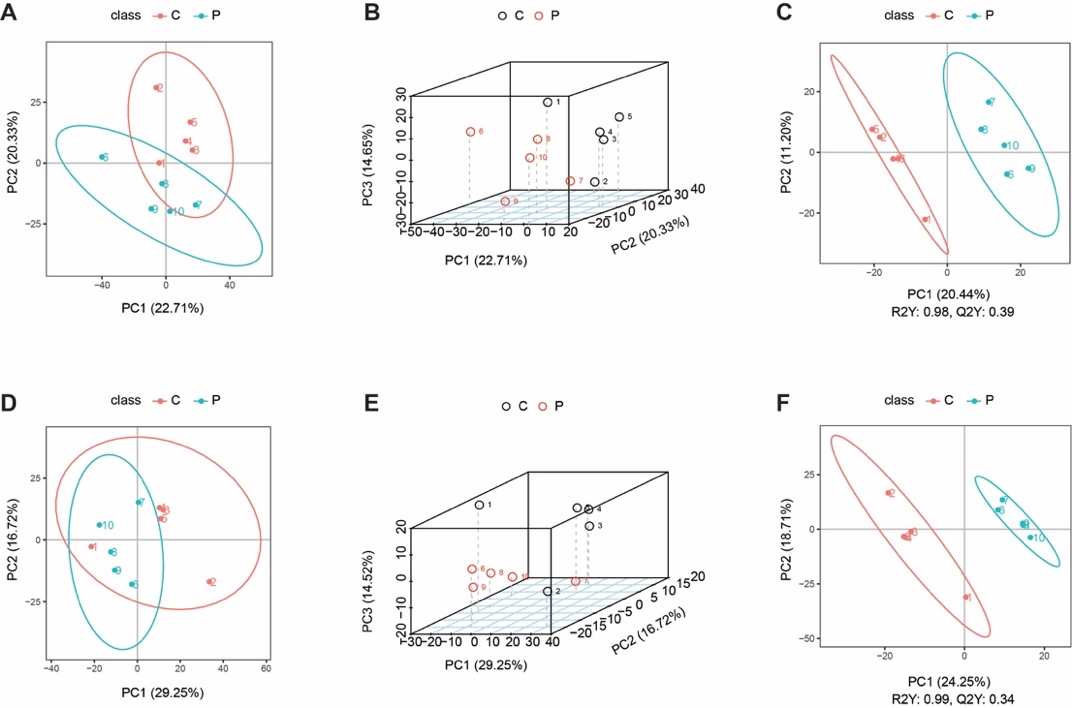


Supplemental Figure 2. **Differential metabolite analysis. *Principal Component Analysis (PCA):*** The abscissa PC1 and ordinate PC2 in the figure represent the scores of the first and second principal components, respectively. The color of the scatter points represents the experimental grouping of the samples, and the confidence ellipsis is 95%. ***Scatter plot and Sort Validation Graph (Partial Least Squares Discrimination Analysis, PLS-DA):*** Scatter plot. The abscissa is the score of the sample on the first principal component. The ordinate is the score of the sample on the second principal component. R2Y represents the interpretation rate of the model, and Q2Y represents the prediction rate of the model. Ranking test, the abscissa represents the correlation between the random group Y and the original group Y, and the ordinate represents the scores of R2 and Q2.

**The upper frame is positive ion mode, and the lower frame is negative ion mode.**
