# Supplementary figures and images for: A comprehensive analysis of metabolomics and transcriptomics to reveal major metabolic pathways and potential biomarkers of human preeclampsia placenta
Source: Front Genet. 2022 Oct 3;13:1010657. doi: 10.3389/fgene.2022.1010657 (PMC9574103; doi:10.3389/fgene.2022.1010657)

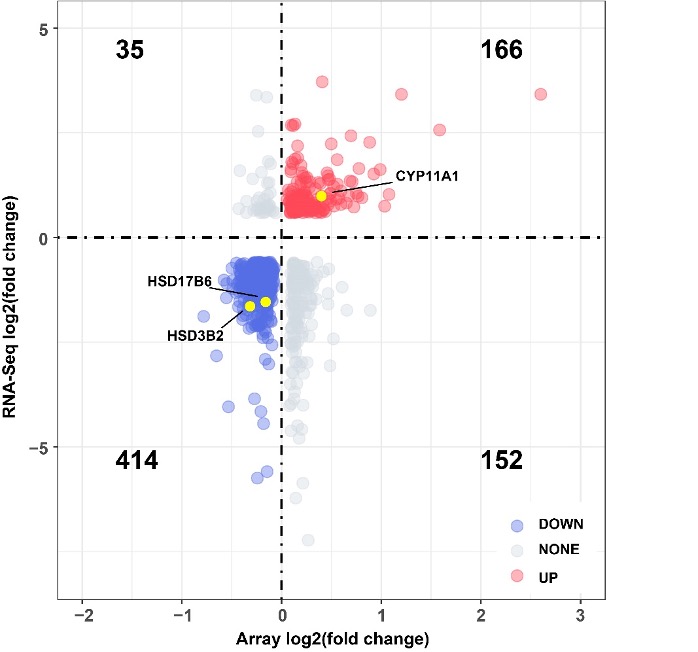

Supplement: Supplementary file 2 [file Image1.JPEG]
